# Informal care-giving and the intention to give up employment: the role of perceived supervisor behaviour in a cohort of German employees

**DOI:** 10.1007/s10433-021-00660-4

**Published:** 2021-11-09

**Authors:** Diego Montano, Richard Peter

**Affiliations:** 1 Department of Population-Based Medicine, Institute of Health Sciences, University of Tübingen, Hoppe-Seyler-Str. 9, 72076 Tübingen, Germany; 2 Department of Medical Sociology, Institute of the History, Philosophy and Ethics of Medicine, Ulm University, Parkstr. 11, 89073 Ulm, Germany

**Keywords:** Informal long-term care, Employment of care-givers, Working conditions, Psychosocial load, Work-private-life conflict

## Abstract

Current social policies in the European Union addressing employment, retirement and long-term care are expected to result in increasing employment rates among informal carers. The present investigation contributes to previous research by focusing on how specific work-related factors, in this case supervisor behaviour, may facilitate the fulfilment of the demands arising from paid work and care and ultimately influence the desire to give up employment. To this end, population data from the German Cohort Study on Work, Age, Health and Work Participation conducted in 2011 and 2014 are analysed (*n* = 3916). Three research hypotheses investigating the associations between care-giving, supervisor behaviour, the intention to give up employment and work-private-life conflict are tested by means of cumulative link models. The results suggest that the intention to give up employment is stronger among employed carers. In addition, the perception that one’s supervisor is considerate towards subordinates and is effective in planning, coordinating work and solving conflicts is found to be negatively related to the desire to give up employment, especially among carers. The statistical analyses reveal that supervisor behaviour mediates the association between the level of work-private-life conflict and the intention to give up employment. It is concluded that the working conditions may exert a substantial influence on the levels of psychosocial load carers experience by facilitating or hindering the extent to which carers are allowed to accommodate their work schedules and job assignments to the conflicting demands of their dual role as workers and carers.

## Introduction

Informal care is defined as care provided by relatives, friends or neighbours, to individuals who have difficulties with performing daily living activities or are in need of permanent nursing care (European Commission 2018). Most informal care recipients are parents, parents-in-law, siblings, spouses or partners, other close relatives and, in general, persons to whom the carer has a strong emotional attachment (Rosa et al. 2011). In Germany, the Statutory Care Insurance Legislation has established that home-care provided by relatives or neighbours is to be preferred to institutional care (Book XI, §3, Code of Social Law SGB). The importance of informal long-term care in Germany is substantial: From the approximately 3.4 million individuals receiving benefits from the German Statutory Care Insurance in 2017, about 1.7 million received informal care from relatives (Statistisches Bundesamt 2017). Estimates obtained from the German Socio-Economic Panel, a large longitudinal household study, revealed that most co-residential care-recipients are the partner or spouse, whereas extra-residential care is provided to parents or parents-in-law (Ehrlich and Kelle 2019). Although care-giving has been related to lower employment rates in the USA and Germany as well (Geyer and Schulz 2014; Lilly et al. 2007), an overall increase in employment rates of carers in Germany has been reported between 2001 and 2012: The employment rates of female and male carers rose from 37 to 61%, and 46% to 67%, respectively (Geyer and Schulz 2014). The proportion of employees providing long-term care in Germany largely increases with age and reaches a maximum among older workers between 45 and 65 years old (Geyer and Schulz 2014).

Within the context of the European Union, employment rates of carers are expected to increase in the coming decades due to several labour market and pension system policies aiming to expand the labour market participation of women, who made up about two-thirds of European carers (Ciani 2012), and to prolong the life time spent in the labour market for both female and male employees (Council Decision of the European Union 2018/1215). Even though home-care and community-based care services are also prioritised in European social policy, long-term care policies largely differ across the Member States of the European Union in important areas such as the eligibility criteria for care benefits, structural organisation of long-term care provision, affordability or the type and covering of available services in the care market (European Commission 2018). Informal care in European states is thus at the intersection of social and labour market policies which, on the one hand, prioritise different forms of informal care and, on the other, indirectly foster the labour market participation of carers.

For a large proportion of employees in their mid-forties, concerns about carer commitments towards relatives such as parents or parents-in-law and the imminence of the decision to retire increase as they become older and, thus, the potential psychosocial load resulting from their dual role as workers and carers may have an impact on their present retirement decisions. Some previous research conducted in the field of vocational psychology has focused on pre- and post-retirement employment decisions of older employees from the perspective of career constructionist and social identity theories focusing on the meaning of work (Fasbender et al. 2015, 2019; Savickas 2020). For instance, career planning for the post-retirement life-phase has been found to be influenced by the occupational future time perspectives (Fasbender et al. 2019), i.e. by the individual’s perceptions of their post-retirement future in the context of employment (Rudolph et al. 2018). Furthermore, the meaning of work, which reflects an appraisal of work in the context of one’s own life (Rosso et al. 2010), seems to increase the likelihood of post-retirement employment among individuals for whom paid work is a source of social activities and personal satisfaction (Fasbender et al. 2015).

Nonetheless, most research findings on care-giving come from studies conducted in the field of social gerontology (Rosa et al. 2011) focusing on the psychosocial load carers experience and their different stress-coping strategies (Carretero et al. 2009). Results from a large survey of carers in six European countries revealed that the most frequent coping strategy of carers to reconcile care-giving and work is to reduce their working hours (Principi et al. 2012). Moreover, with increasing needs of the cared-for person, the likelihood of giving up work increases as well, suggesting that carers’ employment status largely depends also on the perceived level of commitment towards the care-receiver and the level of care needs (Principi et al. 2012). Hence, it seems that the late-career employment decisions depend not only on one’s own attitudes towards work, but also on the social norms determining the individual’s responsibility for caring after relatives who may need support with daily living activities or require permanent nursing care.

Although these previous findings have highlighted the relevance of career expectations, attitudes towards work and social identity as antecedents of the retirement or post-retirement attitudes and decisions of older workers, less is known about specific work-related factors which may help employees reconciling work and carer roles. Hence, the present study contributes to previous research by investigating how specific work-related factors, in this case supervisor behaviour, may influence the decision to give up employment among older employees who provide some form of informal care. To this end, population data from the German Cohort Study on Work, Age, Health and Work Participation (lidA Study) conducted in 2011 (wave 1) and 2014 (wave 2) are analysed.

### Research hypotheses

The intention to retire or give up employment can be conceived as a complex decision-making process involving at least three major antecedent factors: perceptions of self-competence, motivation to continue working, expected socio-psychological rewards from employment, financial and household commitments and the subjective assessment of health functioning (Blanc et al. 2019; Harkonmäki 2006; Wahrendorf et al. 2013; Rudolph et al. 2018). On the other hand, care-giving is a critical life event posing a higher physical and psychosocial load on carers. From the perspective of roles as contextualised patterns of behaviour (Arditi 1987; Biddle 1986), the non-overlapping activities inherent to work and care-giving roles may result in an overload of expectations towards carers. Some of the most important factors determining the amount of psychosocial load in the context of care-giving pertain the quality and type of relationship between care-giver and care-receiver (Braithwaite 1996), the physical and mental demands of care-giving tasks, negative stress and financial hardship (Pearlin et al. 1990). These factors may increase the likelihood of carers reporting lower levels of physical and mental health, and higher levels of depressive symptoms as well (Hiel et al. 2015; del Pino-Casado et al. 2019; Verbakel et al. 2017). Hence, especially employed carers may experience large increases in psychosocial load due to the combination of paid work and care-giving and, thus, a stronger desire to leave employment (Gomez-Leon et al. 2017). Therefore, it is expected that in the lidA Study:

#### Hypothesis 1

Care-giving at wave 1 (W1) is positively associated with the intention to give up employment at wave 2 (W2).

**Fig. 1:**
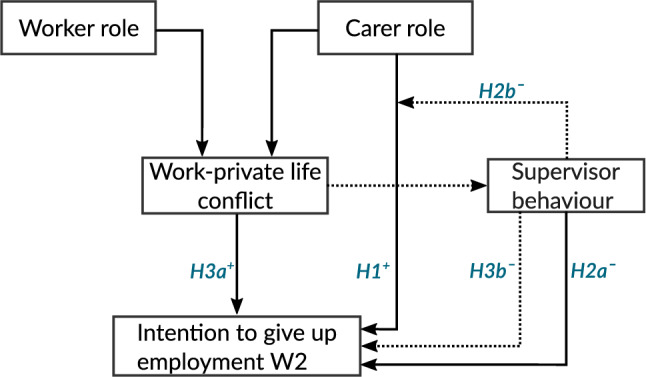
Schematic representation of the theoretical model and the research hypotheses

Furthermore, work characteristics such as job-task definition, formal procedures, organisational rules, psychosocial load, work-related negative stress experiences and social relationships with supervisors and colleagues at the workplace have a strong influence on the intention to retire (Blanc et al. 2019; Harkonmäki 2006; Wahrendorf et al. 2013). Hence, the particular characteristics of the work environment employed carers are faced with have a direct impact on the extent to which employed carers are able to cope with the demands arising from work and care-giving (Grünwald et al. 2020). Supervisor behaviour, as one of the most important factors determining the specific form of working arrangements such as working schedules and rules and procedures, can be expected to have a large influence on how employed care-givers allocate their resources to job and caring activities. In particular, supervisors who are perceived by their subordinates as performing better at planning and organising job tasks, and showing consideration for their subordinates, have been related to higher employee productivity, job satisfaction and improved mental health outcomes in meta-analytic studies (Judge et al. 2004; Montano et al. 2017). In a previous study, employees who believed their supervisors show particular respect and consideration towards their subordinates reported also a lower desire to retire (Wöhrmann et al. 2017). Furthermore, supportive employers seem to moderate the relationship between carers’ perceived stress and work-related outcomes (Kim et al. 2011). Hence, it is hypothesised that:

#### Hypothesis 2

A higher appraisal of supervisor behaviour at W1 is associated (a) with a decreasing intention to give up employment at W2, (b) especially among care-givers.

From a role theoretical perspective (Fig. 1), it has to be taken into account that employed carers are usually entangled in conflicting or contradictory demands in their dual role as carers and workers (Parsons 1951; Arditi 1987; Biddle 1986). The level of perceived conflict between the work and private-life roles has been associated with less work satisfaction, less organisational commitment and increased intention to turnover (Amstad et al. 2011). Moreover, previous meta-analytic findings suggest that low levels of work involvement and job satisfaction are the strongest antecedents affecting the planning and decision to retire (Topa et al. 2009). These results seem to indicate that higher levels of perceived inter-role conflict elicit negative affective and attitudinal states which may ultimately result in a higher intention to give up employment as a form of stress-coping strategy (Lazarus and Folkman 1984) and/or lower self-efficacy expectations concerning one’s own ability to cope with the conflicting demands as carers and workers (Lent and Brown 2013). In this manner, the specific working conditions experienced by care-giving workers are a central factor determining the extent to which both roles can be reconciled, either in the form of facilitating or hindering conditions for the fulfilling of the particular role domain demands. For instance, previous research findings indicate that the inter-role conflict between work and private life mediates the relationship between workplace flexibility characteristics and several work and family outcomes (Brown and Pitt-Catsouphes 2015). Moreover, meta-analytic findings have suggested that supervisor support, especially support towards the reconciliation of work and family commitments, is negatively associated with perceived work-family conflict (Kossek et al. 2011). Therefore, supervisors showing higher skills in planning and organising job tasks and being more considerate towards their subordinates may reduce the inter-role conflicts of work and care-giving demands. Hence, it is expected that:

#### Hypothesis 3

(a) A higher work-private-life conflict at W1 is positively associated with the intention to give up employment at W2, with (b) supervisor behaviour acting as a mediator of that association.

Finally, the inter-role conflict arising from care-giving and work demands has particular gender-specific implications. In a large random sample of carers in six European countries, about 81% of carers were women of whom 47% were employed (Principi et al. 2012). Given the fact that career decisions, occupation, employment and attitudes towards care-giving largely differ between men and women over the life course (Carmichael and Ercolani 2016; Fitzenberger et al. 2004; Guinea-Martin et al. 2018), it can be expected that the perception of supervisor behaviours and the intensity of care-giving activities are to some extent gendered. Hence, in order to capture potential gender effects on the intention to give up employment and to obtain adjusted marginal effect estimates of supervisor behaviour and care-giving, gender is included as a control variable in the investigation of all research hypotheses.

## Methods

### Data and variables

The German Cohort Study on Work, Age, Health and Work Participation (lidA Study) is a population study of employed individuals born in either 1959 or 1965 and subject to social security contributions in Germany (Hasselhorn et al. 2014). The data considered in the present investigation correspond to the first and second lidA waves collected in 2011 and 2014, respectively. Participants were sampled from the “Sample of Integrated Labour Market Biographies” dataset held by the Institute for Employment Research (IAB) (Dorner et al. 2010), which itself is a random sample of all employees subject to social security contributions in Germany. In the present study, only individuals reporting to a supervisor and working full-time, part-time or irregularly employed were considered. The intention to give up employment was measured at wave 1 (W1) and wave 2 (W2) with the single item: “How often in the last 12 months have you had thoughts of completely giving up your employment?” (1: never, 2: a few times a year, 3: a few times a month, 4: a few times per week, 5: every day). Care-giving at W1 was assessed by the dichotomous questions: “Do you provide care to someone in / outside your household?”. Care-giving at W2 was assessed by the dichotomous question: “Do you provide care to someone in your private life?”. Work-private-life conflict at W1 was measured with the following two Likert items translated from the English version of the Copenhagen Psychosocial Questionnaire (COPSOQ) (Cronbach’s $\alpha = 0.87$): “Do you feel that your work drains so much of your energy that it has a negative effect on your private life?” and “Do you feel that your work drains so much of your time that it has a negative effect on your private life?” (1: it does not apply at all, 2: it does not apply, 3: it rather applies; 4: it applies at all) (Kristensen et al. 2005). Respondents’ appraisal of supervisor behaviour was measured with four Likert items from the German COPSOQ version (Nübling et al. 2005) (Cronbach’s $\alpha = 0.83$ and McDonald’s total $\omega _t = 0.83$) (Zinbarg et al. 2005). Respondents assessed the extent to which they believed their supervisor: “makes sure that the individual member of staff has good development opportunities”, “gives high priority to job satisfaction”, “is good at work planning”, “is good at solving problems” (1: to a very large extent, 2: to a large extent, 3: somewhat, 4: to a small extent, 5: to a very small extent). To ease the interpretation of scores, the supervisor scale was reversed so that higher scores indicate a higher appraisal on the supervisor behaviours described by the items.

### Statistical analysis

The research hypotheses were investigated by means of cumulative link models in which the dependent variable is the intention to give up employment at W2. Cumulative link models are an appropriate modelling strategy for this type of ordinal variables (Fahrmeir et al. 2013). Let *Y* be the ordinal variable with $j = 1, \ldots , 5$ categories corresponding to the intention to retire (1: never, 2: a few times a year, 3: a few times a month, 4: a few times per week, 5: every day). The probability of endorsing a certain category $Y = r$ is defined in the cumulative link models as the conditional probability $\pi _{ir} = P(Y = r | X_p), r= 1, 2, \ldots , J$, given a set of covariates $X_p$ for individual *i*. The cumulative logit model with coefficients $\beta$ and the logit distribution $F = \text {logit}$ are defined for a given response category *r* as follows:

1 $$\begin{aligned} \pi _{i, r = 1} = F(\theta _{i, r = 1} - x_{i} \beta ), \qquad \pi _{i, r} = F(\theta _{i r} - x_{i} \beta ) - \pi _{i, r - 1} \end{aligned}$$

with intercept conditions

2 $$\begin{aligned} - \infty = \theta _{i 0}< \theta _{i 1}< \ldots< \theta _{i, r - 1} < \theta _{i r} = \infty \end{aligned}$$

Under this specification, *Y* remains in category *j*, in so far as

3 $$\begin{aligned} \theta _{i , j - 1}< X_{i} \beta + \epsilon < \theta _{i, j} \end{aligned}$$

holds. The regression coefficients reported in the results section correspond to the odds ratios of each independent variable for adjacent categories of the dependent variable, e.g. the odds of endorsing the category “never” against the odds of endorsing “a few times a year”, and similarly for the other categories of the dependent variable. The selection of the independent variables was guided by the research hypotheses described above, after adjusting for gender.

Model 1 investigates Hypothesis 1 by estimating the associations between care-giving at W1 and the intention to give up employment at W2. In model 2 and 3, Hypothesis 2a and 2b are tested by estimating the main effects of supervisor behaviour and the interaction effects with each category of care-giving at W1, respectively. In order to obtain estimates of the interaction terms for each care-giving category and, therefore, ease the comparison of results, the main effects of supervisor behaviour are not included explicitly in Model 3. Please note, however, that the coefficient of the reference category, i.e. no care-giving, is actually equal to the main effect of supervisor behaviour. On the other hand, the investigation of Hypotheses 3a and 3b requires the calculation of mediation effects. In the present study, the product-of-coefficients approach is utilised to obtain estimates for the mediated effect as discussed in MacKinnon et al. (2002). To this end, the following two regression equations are estimated:

4 $$\begin{aligned} P(Y = r | X, I) =&\, F(\theta _{i, r = 1} - (\tau X + \beta I)) \end{aligned}$$

5 $$\begin{aligned} P(I = r | X) =&\, F(\theta _{i, r = 1} - \alpha X) \end{aligned}$$

with the ordinal variables *X* and *I* representing the supervisor behaviour and the intervening variable, i.e. the work-private-life conflict, respectively. To test for the significance of the intervening variable *I* the variance of the coefficient product $\alpha \beta$ is estimated with the unbiased pooled variance estimator $\sigma ^2$ (Goodman 1960):

6 $$\begin{aligned} \sigma ^2 = (\alpha \sigma _{\beta } )^2 + (\beta \sigma _{\alpha } )^2 - (\sigma _{\alpha } \sigma _{\beta })^2, \end{aligned}$$

where $\sigma _{\alpha }, \sigma _{\beta }$ are the standard deviations of the regression coefficients $\alpha , \beta$, respectively. In order to build the corresponding confidence intervals of the coefficient product $\alpha \beta$, the coefficients and their standard deviations are transformed to the *z*-scale of the normal standard distribution by multiplying with $\sqrt{3} / \pi$ as suggested elsewhere (Chinn 2000). Following the simulation results reported by MacKinnon et al. (2002), the quantiles of the confidence intervals are taken from the distribution of the product of two standard normal random variables, in order to reduce type I error rates and obtain more accurate estimates. The mediation effect of supervisor behaviour is stated with the null hypothesis $H_0: \alpha \beta = 0$. In order to ease the interpretation of results, the mediation effect estimates are reported in the odds ratio scale, i.e. $\exp [\alpha \beta \cdot (\sqrt{3}/\pi )^2]$. All statistical analyses are performed with the statistical environment R 3.6.3, especially the package VGAM (Yee 2010).

## Results

A total of 3916 records were obtained with complete data of individuals reporting to a supervisor and working full-time, part-time or irregularly employed (Table 1). Average age of respondents was 48.7 and 51.7 years in the lidA wave 1 (W1) in 2011 and wave 2 (W2) in 2014, respectively. Women were to some extent more frequently engaged in care-giving than males at both W1 (66.5%) and W2 (63.9%). The results pertaining the research hypotheses are reported in Table 2. Regarding Hypothesis 1, the estimates of model 1 confirm that care-giving at W1 is positively associated with the intention to give up employment at W2, with females showing a stronger intention to remain in the labour market.

**Table 1:** Descriptive statistics

Variable	Statistics (%)			
Full-time employed	67.57			
Part-time employed	27.09			
Irregularly employed	5.34			
Males	45.86			
Females	54.14			
No care-giving W1	91.78			
Care-giving W1	8.22			
No care-giving W2	91.45			
Care-giving W2	8.55			

		Correlations		
		Give up employment W1	Give up employment W2	Work-private-life conflict W1
Intention to give up employment W1	1.00 (0.00)			
Intention to give up employment W2	1.00 (0.00)	0.25		
Work-private-life conflict W1	2.00 (0.74)	0.09	0.14	
Task-oriented leadership W1	3.20 (1.04)	− 0.13	− 0.08	− 0.21

Percent values for categorical variables. Median and median absolute deviation in parentheses for ordinal variables. $N = 3916$. Spearman correlations

**Fig. 2:**
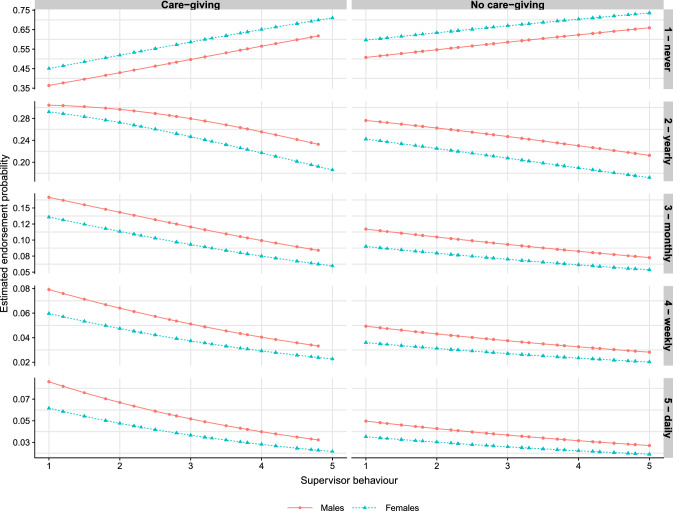
Estimated endorsement probabilities to the intention to give up employment at W2 by levels of supervisor behaviour (*x*-axis, 1: to a very large extent, 2: to a large extent, 3: somewhat, 4: to a small extent, 5: to a very small extent), care-giving status (columns) and gender. Estimates obtained from model 3 in Table 2

In agreement with Hypothesis 2a, higher scores of supervisor behaviour at W1 were related to lower odds of the intention to give up employment at W2 (model 2). Similarly, for Hypothesis 2b, the estimates of model 3 concerning the interaction effect between care-giving and supervisor behaviour indicate that the supervisor effects are slightly larger among carers in comparison to non-carers (0.76 95% CI [0.62–0.94] vs. 0.85 95% CI [0.79–0.92], respectively). In order to ease the interpretation of the coefficients in model 3, the panels in Fig. 2 depict the changes of the estimated endorsement probabilities for each answer category of the intention to give up employment at W2, according to whether the respondents provide care or not, and by the levels of the perceived supervisor behaviour scale. It can be observed in Fig. 2 that the estimated proportion of employees never having thought on giving up employment at W2 (first row of panels) increases with higher levels of supervisor behaviour. For the remaining answer categories of the intention to give up employment at W2 (rows 2 to 5 in Fig. 2), the results indicate that higher supervisor behaviour scores are associated with a decreased intention to give up employment. Notice that the curvature of the lines corresponding to the interaction effects in Fig. 2 is somewhat more pronounced among the carers than among the non-carers. This result suggests that the association between the intention to give up employment at W2 and supervisor behaviour is somewhat stronger among carers (Table 2). The differences between males and females concerning the endorsement probabilities are substantial due to the fact that males tend to think much more often about giving up employment than females for each answer category. In particular, males reporting the lowest scores of supervisor behaviour were found to think a few times a week or daily on giving up employment, especially among carers (Fig. 2).

**Table 2:** Cumulative link models

Variable	Model 1	Model 2	Model 3	Model 4
Intercept 1	0.69 [0.63–0.76]	1.21 [0.95–1.52]	1.14 [0.89–1.45]	0.50 [0.36–0.68]
Intercept 2	0.20 [0.18–0.22]	0.34 [0.27–0.44]	0.32 [0.25–0.41]	0.14 [0.10–0.19]
Intercept 3	0.08 [0.07–0.09]	0.14 [0.11–0.18]	0.13 [0.10–0.17]	0.06 [0.04–0.08]
Intercept 4	0.04 [0.03–0.05]	0.07 [0.05–0.09]	0.06 [0.05–0.08]	0.03 [0.02–0.04]
Females	0.69 [0.61–0.78]	0.71 [0.62–0.81]	0.70 [0.61–0.79]	0.74 [0.65–0.84]
Care-giving W1 (ref. no)	1.38 [1.11–1.72]		2.02 [0.98–4.17]	
Supervisor’s behaviour		0.84 [0.79–0.90]		0.89 [0.83–0.96]
Care-giving (yes) $\times$ Supervisor			0.85 [0.79–0.92]	
Care-giving (no) $\times$ Supervisor			0.76 [0.62–0.94]	
Work-private-life conflict				1.39 [1.29–1.50]
AIC	8445	8181	8171	8111
$R^2_{\mathrm{Nagelkerke}}$	0.01	0.09	0.09	0.10

Dependent variable: intention to give up employment at W2. Odds ratio and 95% confidence intervals. *AIC* Akaike’s Information Criterion. *n* = 3916

In model 4, the odds ratio estimate of the work-private-life conflict suggests that the intention to give up employment increases with higher levels of perceived inter-role conflict, thereby confirming Hypothesis 3a. Concerning the mediation analysis, the product-of-coefficients of supervisor behaviour and work-private-life conflict on the odds ratio scale yielded $\alpha \beta = 0.93$ 95% CI [0.86; 0.97], ($\alpha =-0.41$, $\sigma _{\alpha } = 0.03$, $\beta =0.33$ and $\sigma _{\beta }= 0.04$), i.e. supervisor behaviour seems to mitigate the negative association between work-life-private-life conflict and the intention to give up employment by a factor of 0.93 on the odds ratio scale, thereby providing support for Hypothesis 3b.

## Discussion

The results provide support for the three research hypotheses investigated in the present study (Fig. 1). It was found that the intention to give up employment is stronger among employed carers in comparison to non-carers. In addition, the analysis revealed that perceiving one’s supervisor as having sufficient skills to plan and coordinate work and showing consideration for employees is negatively related to the desire to give up employment. Moreover, the estimates suggest that the influence of supervisor behaviour on the desire to give up employment may be even stronger among employed carers. The results provided also support to the notion that a more intense experience of inter-role conflict between private life and work demands increases the desire of giving up employment. At the same time, however, evidence was found that supervisor behaviour may reduce the perceived inter-role conflict and contribute to a reduction of the intention to give up employment. Given the particular relevance the findings of the present study have for work organisations, we would like to elaborate on some important organisational implications in regard to the role of supervisors and to ageing and retirement behaviour.

### Implications for the work environment

The present findings emphasise that the day-to-day social aspects of work, in this case, the experience of how supervisors behave, may be particularly important for carers. These results extend the observations made in previous studies focusing on particular aspects of the work environment such as employers’ explicit informal support towards carers (Kim et al. 2011), working time arrangements or workplace health support (Grünwald et al. 2020). In this study, it was found that supervisors who show consideration towards subordinates, reduce work conflicts and who are perceived as particularly effective in planning and organising work, may exert a substantial positive influence on the subordinates decision to remain in the labour market, especially among carers. These observations can be partly explained by (1) organisational factors related to how labour is coordinated in organisations, and the ways how resources and power are allocated, and (2) socio-psychological factors related to supervisor support and consideration.

First, among the organisational factors, qualitative research has indicated that employed carers are particularly affected by challenges related to the prioritisation of work tasks, negotiation of deadlines and allocation of job tasks with colleagues and supervisors, adjustment of working hours and schedules and the need of taking work home (Gaugler et al. 2018; Scharlach 1994). Supervisors who are effective in planning and coordinating work may be more able to adjust work processes within and between organisational units and, therefore, help carers accomplish both work and care demands. Given the fact that supervisors have a higher influence on the power hierarchy within organisations, they also have the authority to re-allocate job assignments and resources between and within work groups or facilitate the negotiation of task prioritisation in conflicting work situations (Montano 2016). Second, from a socio-psychological perspective, supervisors who prioritise employees’ job satisfaction and development opportunities implicitly show consideration towards the well-being of employees, and consequently, they may be more aware of the emotional states of subordinates (Humphrey 2002; Kaplan et al. 2014). Hence, such supervisors may be more willing to provide adequate support to carers.

Notice that the work-private-life conflict in this study actually represents the respondents’ appraisals of the extent to which work drains their time and energy. Accordingly, the observed mediation effect of supervisor behaviour on the work-private-life conflict may be an indication that supervisors may effectively reduce the workload and facilitate an appropriate allocation of time between work and private life. A reduction of the work-private-life conflict seems to be particularly beneficial among carers who are often challenged by unpredicted and conflicting demands arising in the context of their dual role as employees and carers. For instance, a previous study with employees between ages 50 and 55 in the UK found that the intensification of care increases the chances of carers leaving the labour market (Gomez-Leon et al. 2017). Hence, the degree of adaptability of carers to changing role demands would depend on whether supervisors make possible a situational re-accommodation of resource allocation between work and private life. Since employed carers and non-carers seem to differ above all in the number of hours committed to work (Lilly et al. 2007), organisations aiming to retain their work force would likely be more successful by ensuring via supervisor behaviour that the workload and the working time arrangements are to some extent commensurate to the carers’ needs. The observation that males think more often about giving up employment suggests that male carers may experience more difficulties in reconciling their dual roles as carers and workers. Thus, gender-specific work arrangements are likely to be more effective than generic ones in facilitating the reconciliation of carer and worker roles.

### Ageing and retirement behaviour

From an ageing perspective, the results have some important implications. It should be recalled that the lidA cohort is composed of adults between 46 and 55 at the time of data collection among whom care commitments towards relatives, usually parents and parents-in-law, are most likely to occur. However, only about 8% of participants in the lidA cohort aged 46–55 provided informal care, suggesting that several factors account for the amount of care family members expend. For instance, the availability and utilisation of care services, the health status and the degree of self-sufficiency of relatives may contribute to the determination of the amount of care demands required from the middle-aged population (Settersten 2007). In addition, despite the fact that individuals between 46 and 55 years usually experience a peak of care, family and work demands, findings from a previous study from the USA have suggested that filial responsibility for assisting older parents begin to weaken at the start of middle age and further decline with increasing age (Gans and Silverstein 2006). This would imply that particularly middle-aged individuals with a high sense of filial responsibility may be more inclined to engage in care-giving. This seems to be supported by a previous study with a German sample in which one of the best predictors of post-retirement family care was the personal commitment to sharing knowledge to the next generation and a high sense of contributing to society (Fasbender et al. 2015), i.e. individuals with a high sense of altruistic attitudes towards society in general, and family in particular.

Although the datasets used in the present investigation do not allow a detailed account of actual retirement behaviour, the intention to give up employment can be considered as a proxy of the intention to retire for this cohort of German employees age 46 years and older, for whom alternative forms of economic activity such as self-employment or entrepreneurship are far less relevant. As previous research has indicated, the intention to retire can be a strong predictor of actual retirement behaviour (Harkonmäki et al. 2009). Even though it seems that the strongest effect is related to the preference for a specific retirement age (Solem et al. 2014), the intention to retire captures aspects related to the employees’ assessments of their own motivational state and self-efficacy expectations (Bandura 1986), which may be conducive to observed retirement behaviours. The finding that carers have a stronger desire to give up employment may partly explain previous results from Germany with data from the Socio-Economic Panel Study suggesting that the probability of retirement is higher for carers in comparison to non-carers (Meng 2012).

### Limitations

An important limitation of the present investigation is that the datasets of the lidA Cohort Study do not include specific information on the care-giving situation, and therefore, more detailed analyses are not feasible. Data collection on care-giving is limited to “nursing”, especially of ill or disabled persons, albeit this connotation is rather implicit in the original German items (see “Methods” section). Hence, the prevalence rates of care-giving reported in the present study cannot be directly compared to those obtained in other population studies with broader definitions of care-giving such as the European Social Survey, in which respondents are asked whether they spend any time looking after or giving help to family members, friends, neighbours or others because of illness, disability or problems related to old age (Verbakel et al. 2017). Moreover, the present investigation focuses on the extent to which care-giving, supervisor behaviour and the potential ensuing inter-role conflict affect the intention to give up employment. Hence, the investigation of further factors determining the decision to retire or continue employment such as income, occupation and labour-market opportunities could not be pursued in the analyses. Future research may consider the inclusion of additional socio-demographic and labour-market characteristics of carers and their interaction with the care-giving situation.

## Conclusions

Care-giving is associated with an increased desire to completely give up employment among employed carers in Germany. The intention to give up employment was lower among employees who perceive their supervisors as considerate and effective in planning, coordinating work and solving conflicts at the workplace, especially employed carers. Furthermore, supervisor behaviour seems to mediate the associations between the experienced work-private-life conflict and the intention to give up employment. Therefore, the working conditions in general, and the enacted supervisor behaviour in particular, may exert a substantial influence on the levels of psychosocial load carers experience by facilitating or hindering the extent to which carers are allowed to accommodate their work schedules and job assignments to the conflicting demands of their dual role as workers and carers.
